# Regional wood chip quality parameters decomposition and price linkage with impact on Polish energy sustainability: Time frequency analysis between 2013 and 2019

**DOI:** 10.1016/j.heliyon.2024.e33322

**Published:** 2024-06-20

**Authors:** Michal Čermák, Jitka Malaťáková, Jan Malaťák, Monika Aniszewska, Arkadiusz Gendek

**Affiliations:** a Department of Eonomics, Faculty of Economics and Management, Czech University of Life Sciences Prague, Kamýcká 129, 165 21, Prague Czech Republic; b Department of Technological Equipment of Buildings, Faculty of Engineering, Czech University of Life Sciences Prague, Kamýcká 129, 165 21 Prague, Czech Republic; c Department of Biosystems Engineering, Institute of Mechanical Engineering, Warsaw Uniwersity of Life Sciences, Nowoursynowska 164, 02-787 Warszawa, Poland

**Keywords:** Wood chips, Wood chip price, Calorific value, Moisture content, Power plant, Wavelet coherence, Wavelet transform

## Abstract

The study aims to analyze and decompose the qualitative parameters of wood chips in Poland. The European Green Deal brings the new framework to support sustainability and elimination of emissions. The Wavelet coherence and Wavelet Discrete Decomposition are used for determination of relations among significant qualitative parameters. Thus, the possible uses are discussed. For the obvious relationship between moisture and calorific value there is evidence of strong correlation. The behaviour of these interrelations are different at frequencies in the long and short time. The wood chip price is inter-transmitter from moisture parameter to calorific value in a positive (in-phase) relationship. At both low and high frequencies there is evidence that the variables of moisture and calorific value are highly correlated. The transient effect of linkage is presented at values between 0.1 and 0.3 in coherence map. The empirical findings provide implication for local producers and policymakers.

## Highlights

• The wood chips qualitative parameters in Polish region are analyzed.
• Wavelet coherence and DWT decomposition are employed.
• The strong correlation between moisture and calorific value of wood chips.
• The evidence of stronger correlation between price of wood chip, calorific value, and moisture than ash content.

## Graphical abstract

**Figure:**
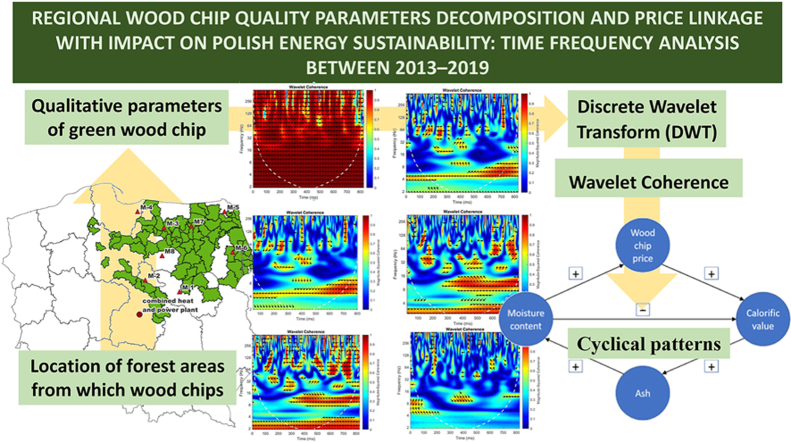

## 1 Introduction

There is increasing pressure globally to reduce the consumption of coal, which is currently the main source used to generate heat and power, but also a source of greenhouse gases [1]. China is the largest CO_2_ emitter and consumer of coal [[2], [3]]. Other large CO_2_ producers include the United States, India, Australia, Indonesia, Russia, South Africa, Germany, and Poland [[4], [5], [6]]. One pressing question is how to replace these fossil energy sources [[7], [8]]. One possibility is to use energy from renewable biological sources in the form of wood chips [9].

The share of coal in the total electricity generation in the EU is still high, around 20 %. Coal power also provides employment for around 230,000 people in mines and power plants in 31 regions and 11 EU countries [10]. Climate and climate change are generally influenced by many factors, including vigorous activities such as burning fossil fuels, deforestation, nitrogen fertilization, fluorine-based gases, and animal husbandry [[11], [12]]. According to National Energy & Climate Change [13], power plants and cogeneration plants had the largest share of energy production in Poland in 2021, burning mainly hard coal (about 50 %) and lignite (about 26 %), natural gas (about 7.5 %), oil (1.3 %), and biomass (1.2 %) [14]. The use of renewables in Poland is very low compared to EU countries [15,16]. At the beginning of January 2021, the total installed electricity capacity was 45.029 GW. This is sufficient capacity to meet national needs [17]. Most of the power is generated by thermal power plants using coal and lignite (U.S [18]). Of the 18 power plants in Poland, only 7 use lignite for power generation, with the lignite plant with the largest installed capacity (5 GWe) being the Bełchatów plant [19]. The remaining 11 power plants use hard coal. Recently, several power plants have undergone upgrades [20], through which biomass burned in dedicated plants or co-firing with coal [21] is used for power generation [22].

Green energy in Poland is mainly wind farms [23], which contributed more than 9.0 % to electricity production, and other renewable sources more than 4,0 % (including water 1.1 %, photovoltaics 2.9 %) [24]. In Poland, the main sources of woody biomass are forestry and the wood and paper industry [[25], [26], [27]]. From 2018 to 2020, approximately 7.5 million m^3^ of wood used for energy production was harvested annually in Polish forests, destined for both individual consumers and industry [28]. Currently, this value represents almost 18 % of the total timber harvest [29].

Between 2004 and 2020, consumption of woody biomass for energy production increased from 13.8 million m^3^ to 23.4 million m^3^ [26]. Between 2005 and 2020, the bioenergy sector in Poland developed dynamically [30]. The total capacity of biomass plants increased sevenfold (697 %) from less than 190 MW to 1512 MW [29]. The amount of woody biomass consumed in bioenergy increased almost 140 times (13.852 %) from 35,000 m^3^ to 4.9 million m^3^ per year in the same period [31].

In 2019, woody biomass combustion was responsible for 52 % of primary energy consumption from RES and 5 % of total primary energy consumption in Poland [32]. In 2020, 7.5 million m^3^ of wood used for energy production was harvested, accounting for 18 % of the total wood harvest in Polish forests [33]. Currently, there are 21 RES installations in Poland with in with an installed capacity higher than 5 MW powered by biomass, which burns woody biomass [34]. The largest installations consume more than 500,000 tonnes of forest chips each year [29].

According to the Statistical Yearbook of Forestry of the Central Statistical Office [35], the harvest of firewood in 2022 alone amounted to 2.958 million m^3^. The estimated harvest of energy wood this year was about 7.5 million m^3^, more than double. The reason for the discrepancy in the data may be the fact that the State Forest Holding State Forests (PGL LP), the largest supplier of timber to the domestic market (over 90 % of timber harvest), has no influence on the allocation of raw material purchased from it. State Forests does not sell timber directly to energy companies. Biomass suppliers to large energy companies are companies specializing in biomass trading. Forest chips are the main fuel in biomass burning plants. In 2018, LP sold only 255 thousand m^3^ of wood chips 16.6 thousand m^3^ in 2019, and 0 m^3^ in the following years. In 2018–2021, the State Forests sold 1.58–1.74 million m^3^ of small-scale timber, an unspecified amount of which was chipped and sold to the energy sector [36].

The recognition of woody biomass as a renewable energy source in 2009 led to increased imports of wood for energy purposes into Poland [37]. In 2010, 0.21 million tonnes (Mt) of woody biomass for energy purposes was imported to Poland and in 2020 it was already 2.19 Mt. Biomass was imported to Poland mainly from Belarus, Ukraine and Russia and exported to Western European countries (about 50 % to Germany) [29].

The quality of the wood chips plays a major role in the selection of the appropriate energy recovery method such as direct combustion [38], gasification [39] and pyrolysis treatment [40]. The direct combustion of biomass is influenced by both the form and quality of these wood chips [41], pellets [42] and briquettes [43]. The elemental composition of the fuel is essential for the dynamics of the combustion process [44]. The proportion of the non-flammable part of the fuel such as moisture and ash form the technical requirements for the combustion equipment [45]. The proportion of the combustible part of the fuel forms the process requirements for the combustion process to ensure perfect combustion of the fuel [[46], [47]]. Moisture content in woody biomass significantly affects the overall calorific value [48,49]. In contrast to the ash content of wood biomass, the moisture content can be optimized according to storage conditions, such as storage method [50,51], or the size of the stored chips [52]. Reduction of moisture content is necessary for thermochemical processes [39,53]. In contrast to the other biomass, the ash content of woody biomass is very low and depends on the origin of the wood [54]. The lowest ash content is found in pure woody biomass, where the sulfur content can be below 0.5 wt% [44]. The ash content, in contrast to the moisture content, due to the low ash concentration, does not have such a significant effect on the overall calorific value of woody biomass [48] and is primarily a source of technical problems in the actual thermochemical processing [53,55].

These all parameters must be in accordance with Fit for 55 [56] and the European Green Deal. The European Green Deal defines a strategy for a sustainable environmental roadmap to 2050. The plan (EGD) has set itself the objective of acting to manage climate change. The main objective of the EGD is to act on the transformation of the European economy and society to a more sustainable trend [57] The GHG emissions within Poland represent about 8.5 % of the total EU-27 production [58]. In 2019, the average per capita carbon dioxin emission in Poland was 10.4 t. This value was still above the EU-27 average. Historically, between 2005 and 2019, GHG emissions in Poland decreased by only 2.3 % (the EU average was a 22 % decrease) [59].

The carbon intensity of the Polish economy is 172 % above the EU average [60]. This places Poland in second place within the EU member states. During this period, the Polish economy grew by about 1–7 % per annum. The volume of emissions remained at the same level during this period. The energy sector was the largest emitter in 2019, accounting for 38 % of Poland's total emissions [61,62]. Poland has the largest coal and lignite reserves in the EU[63]. In 2019, approximately 40 % of energy was covered by coal. In 2020, almost 70 % of Poland's electricity came from coal [29].

. The wavelet coherence method was used to analyze the integration of stock markets. As an extension of traditional wavelet coherence, a new method of analyzing spillover effects based on discrete wavelet transform was also used. This method is more applicable to capture the time-variation process. The wavelet approach (regarding Discrete wavelet transform and Wavelet coherence) is a powerful tool for analysis of interconnectedness and sensitivity of qualitative parameters.

The paper focuses on a time-frequency analysis of the quality parameters of wood chips in a major Polish region. Potential applications for reducing carbon intensity in Poland are discussed. The analysis formulates the research question.

Q1 Are the qualitative parameters interdependent on each other using the example of the Polish region?

The confirmation of this research hypothesis may open the space for gradual substitution of fossil fuel use in the Polish energy market. The aim of the paper is to evaluate the interconnectedness in terms of quality of wood chips and price fluctuations based on monthly frequency. The methodological basis is a wavelet approach to detect time-frequency changes in quality and price values. The empirical analysis discusses the applicability to a possible transition to biomass energy within the framework of the implementation of the Green Plan in Poland.

Therefore, the research focuses on the analysis of time-frequency interrelationships between qualitative parameters or variables, where the course of the COVID-19 pandemic is not considered, as it is assumed that the skew in how the value growth of the Polish economy returns to the trend in the pre-pandemic period.

The remainder of the paper is organized as follows. The second section Materials and Methods briefly provides a description of data used for research and wavelet approaches. The third chapter discusses the main empirical findings, signal decomposition and wavelet coherence. The fourth chapter is focused on discussi the main findings. The next chapter provides a brief discussion on policy implication for policymakers and local governments. The last chapter concludes all remarks of the study.

## 2 Materials and Methods

### 2.1 Harvesting of wood chips

**Fig. 1:**
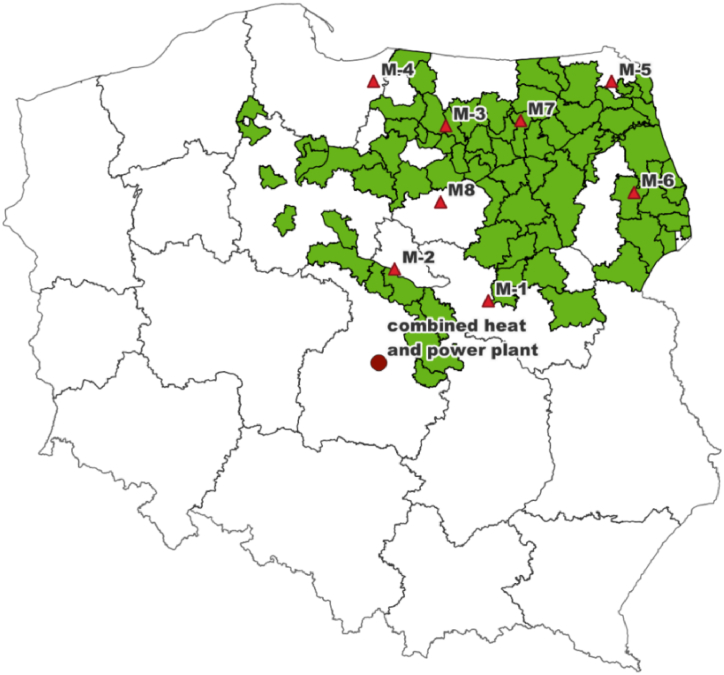
Location of forest areas from which wood chips were extracted, location of the power plant and meteorological measuring points (M1 – Warsaw, M2 – Płock, M3 – Olsztyn, M4 – Elbląg, M5 – Suwałki, M6 – Białystok, M7 – Mikołajki, M8 – Mława).

For the actual analysis, the most commonly used woody biomass in the form of green wood chips was selected [48]. The wood chips came from forest stands in northeastern Poland, see Fig. 1. Polish forests are dominated by coniferous species, also in the north-eastern region of Poland, where wood supplied to energy plants was harvested and chipped.

The ash content of forest chips varies and depends on a number of factors such as e.g. chip composition, proportion of mineral impurities. According to the literature, the amount of ash in woodchips ranges from about 0.5 % for pure woodchips to about 5–7% for woodchips from logging residues, which are often contaminated. The average amount of ash in forest chips is usually in the range of 2–5%. Log residues were crushed, which included tree crowns, branches, needles, leaves, bark and undersized pieces of wood that did not meet the quality standards according to the Quality and Dimensional Grading Order of the Director General of State Forests No. 51 of September 30, 2019 (No.: ZM.800.8.2019) on the introduction of technical conditions for trading in timber raw material in the National Forestry of the State Forests of Poland. Depending on the treatment, the remains came from felling or pre-logging. The species composition of wood chips is diverse, the main species being pine, the share of which can be estimated at more than 70 % [64]. The remaining admixture was spruce and broadleaf species. This composition is related to the dominant species in the area and in Polish forests. Chipping was carried out by a company specialized in the production of forest chips for energy purposes, which has 9 Bruks 805CT chippers and more than 35 tractor-trailers for transporting wood chips. Crushing of the residues was carried out directly on the harvesting areas or after piling in heaps in the roads adjacent to the harvesting areas. Chipping residues usually lay on the forest surface for several months, naturally losing some of the moisture contained in them [65]. The technologies applied in the study plots for the chipping of logging residues and their transport of woodchips to energy plants were described by Moskalik and Gendek [66], and Gendek et al [67]. The timing of the start of the work depended largely on the date of the timber sale by the forest district and on provisions in the contracts that respected the heating season in the area.

However, this section does not describe the entire process of handling raw material for testing. The terminology presented includes several assortments, some of which are not stated correctly or do not reflect the commonly used terminology in the field. Similarly, the terminology for the production of woodchips includes a mixture of several technically different methods (crushing, shredding, grinding). Important details on the processing of the raw material, e.g. storage, are missing. The second section focuses on the quality parameters of woodchips. This section lacks details on the handling of the material, e.g. transport and storage.

### 2.2 Quality parameters of wood chips

The wood chips produced from the forest area were transported directly to a heating plant belonging to the Veolia Energia Polska network, located in one of the large cities in central Poland (GPS: N51.746644, E19.538882) or, mainly in summer, to a wood chip warehouse located approximately 20 km from the CHP unit. In the case of transport to the landfill, after unloading (usually in the summer), the wood chips were stored in stacks in an open area for up to approx. 2–4 months and then transported to the power plant. The CHP unit operates on 8 boilers with a thermal output of 804 MW and an electrical output of 205.85 MW. In 2022, 80.64 % of hard coal and 19.12 % of renewable energy sources, including biomass, were used to generate electricity and 83.78 % and 14.81 % were used to generate thermal energy [68].

Among the significant costs are the transportation distance of wood chips. The transport distance to the depot or power plant, depending on the location of the forest areas, is in the range of 200–350 km. These transportation costs are analyzed in the papers by Gendek and Nurek [69] and Gendek et al. [67]. Transportation distances are not analyzed in this paper.

The qualitative parameters of green wood chips are assessed for basic fuel parameters [[46], [70]]. Before unloading at the power plant, samples were taken from each transport to determine the average moisture content, calorific value, sulfur content and ash content. Due to the trace amounts of sulfur in the analyzed woody biomass samples, which is also achieved in similar works [[44], [47]], the amount of sulfur is not further analyzed in this paper. These parameters were averaged over all deliveries from a single transport day. The average moisture values are based on the standards for average moisture ISO 18134–3:2015 [71], ash content ISO 18122:2022 [72], calorific value and heat of combustion ISO 1928:2009 [73] and conversion of analytical results according to ISO 16993:2016 [74]. The measurements were performed in the laboratory of the energy company, which issued a biomass acceptance report to the supplier, describing the measured values and specifying the fulfillment of the requirements specified in the contract.

Information on weather conditions in the area of the shipping company's operations was downloaded from the database of the Institute of Meteorology and Water Management – National Research Institute [75]. Eight points were determined (Fig. 1) for which the daily average air temperature (DAT, °C), daily average humidity (RH, %) and daily precipitation (YRR, mm m^−2^) were taken for each day in the period from August 1, 2013 to November 1, 2019. The average values were determined for the analyses for the whole region.

The measured data on wood chip energy parameters, their price and weather conditions were subjected to statistical analysis using Statistica v.13 software. The qualitative parameters of wood chips obtained in this way are further assessed for the elimination of carbon intensity in Poland.

### 2.3 Time-frequency methodology

In this study, we analyzed the connectedness of qualitative parameters of wood chips on the one side and price of wood chips on the other side for sensitivity and phase causality purposes. This approach allows to analyze of variables in the time-frequency domain. The discrete wavelet transform and wavelet coherence are briefly described below.

The Discrete Wavelet Transform (DWT) uses a process, which transforms the time series of variable into different coefficients and vectors. For the purpose of the research is used a Daubechies type of function. DWT is defined as:

(1) $$DWT\left(m,t\right)=\frac{1}{\sqrt{a^{m}}}\sum x\left(n\right)f\left(\frac{t-nba^{m}}{a^{m}}\right)\text{,}$$

where *m* and t are integer parameters, which refers to *a* and *b* scaling functions, the parameter *t* relates to input signal; *n* represents time index in discrete form; *f(t)* is mother wavelet and *x(t)* is signal of time series process, see Pharmaj, Bhardwaj.

The investigated spectral transformations are based on wavelet analysis according to Daubechies, for more details see wavelet decomposition techniques. Thus, the wavelet decomposition is expressed by a third level decomposition: $s=a_{3}+d_{3}+d_{2}+d_{1}$, where s represents the time series, $a_{3}$ the approximation of the given signal, and $d_{3},d_{2},d_{1}$ are then the individual components (coefficients) of the time scale.

Wavelet coherence, which has become popular in finance and economics, is used for frequency and time analysis. This model captures the interactions between different time series and their evolution in time and frequency space. Wavelet coherence is effective in identifying regions with higher common movements in time-frequency space, see for example Kristoufek et al. [76] and Muchebve et al. [77]. Another advantage of using this method is in capturing leading lags in the nonlinear relationship between time series. According to Cohen [78], the use of wavelet analysis is the most widely used method to handle non-stationary time series, which is the case for financial time series of commodity prices. The wavelet transform is filtered into different segments in the time and frequency domain.

Wavelet analysis has been used extensively by many researchers in recent years, see ([[79], [80], [81]] or [82]). All existing research has focused on the use of statistical techniques to capture the relationship between two variables that are represented by a time series.

Wavelet coherence ${\;W}_{\left(s,n\right)}t$ is defined as:

(2) $${\;W}_{\left(s,n\right)}t=\frac{1}{\sqrt{n}}\int_{-\infty }^{\infty }x\left(t\right)b^{*}\left(\frac{t-s}{n}\right)dt\text{,}$$

where n is a scaling parameter and s is a variable parameter, the variable *x(t)* is the so-called analyzed wave. In equation (2), the expression 1n is used to provide the power of the energy. Where n is the scaling parameter and s is a variable parameter, the variable *x(t)* is the so-called analyzed wave. The variable $b^{*}$ is then the complex time element of the wave function *b(t)* itself. This variable is effective in providing stability in both time dimensions. The most well-known type of wavelet analysis is the Morlet wavelet transform.

The cross-wavelet transform (CWT) of two time series *x(t)* and *y(t)* defined by Torrence and Compo [83] with the wavelet transforms $W_{n}^{x}\left(u,s\right)$ and $W_{n}^{v}\left(u,s\right)$ specified in equation (3): with the effect of frequency analysis. The variable b* is then the complex time element of the wave function b(t) itself. This variable is effective in providing stability in both time dimensions. The most well-known type of wavelet analysis is the Morlet wavelet transform.

A cross-wavelet transform (CWT) of two time series x(t) and y(t) defined by Torrence and Compo [83] with wavelet transforms $W_{n}^{x}\;\left(u,s\right)$ and $W_{n}^{v}\left(u,s\right)$ specified in equation (3):

(3) $$W_{x,y\;}\left(u,s\right)=W_{x}\left(u,s\right)W_{y}^{*}\left(u,s\right)$$

Where W_x_ (u,s) and W_y_* (u,s)are the continuous wavelet transforms of the two time series x(t) and y(t); u is then the position index s is then the scaling parameter and the * symbol shows the complex timer. The wavelet transform assumes local covariance between the two variables.

The strength of the cross-wave relationship determines the regions where there is significant correlation between the X(t) and Y(t) variables [84]. The wavelet coherence method is assumed to remain robust to the measurement of the cross-wavelet relationship with the display of significant covariances at each scale of the time series [85]. Extending the work of Torrence and Webster [86], the equation is then defined by including the squares of the wavelet coefficients:

(4) $$R^{2}\left(u,s\right)=\frac{{\left|S\left(s^{-1}W_{x,y}\left(u,s\right)\right)\right|}^{2}}{S\left(s^{-1}{\left|W_{x}\left(u,s\right)\right|}^{2}\right)\;S\left(s^{-1}{\left|W_{y}\left(u,s\right)\right|}^{2}\right)}\text{,}$$

where the parameter s is the space-time operator, and the wavelet coefficient of squares then takes values 0 ≤ R^2^ (u,s)≤1. This is the classical correlation coefficient without smoothing coherence, in this case equal to 1. The smoothing operator *s* in the time and frequency dimensions is then:

(5) $$S\left(W\right)=S_{scale}\left(S_{t}\left(w_{n}\left(s\right)\right)\right)$$

Where S_scale_ is the smoothing of the wavelet scale and S_t_ is the smoothing through time. To detect higher co-movement between two time series, then the value of the wavelet coherence squares R^2^ (u,s) is also higher and vice versa.

The problem of the coefficient of wavelet coherence of squares is taking values in the positive sense in the interval (0–1). This problem makes it impossible to observe differences between positive and negative co-movements of the time series. To overcome this problem, Grinsted et al. [84] and Torrence, Compo [87] proposed the use of phase differences. Thus, the difference between positive and negative movements is already captured. For phase differencing the relationship between the two-time series, the following equation is then defined: (see authors above):

(6) $$\beta^{x,y}=arctg\;\left[\frac{Im\left(W^{x,y}\left(s,n\right)\right)}{Re\left(W^{x,y}\left(s,n\right)\right)}\right]$$

Where $Im\left(W^{\left(x,y\right)}\right)$ and $Re\left(W^{\left(x,y\right)}\right)$ are the imaginary and real components of the smoothed cross-wavelet transform. The phase variable β(x,y)ϵ(0;π2) determines that the second variable (y) is lagged with respect to the first variable (x). Conversely, the relation β(x,y)ϵ(0;−π2) determines that the first variable (x) is lagged, whereas the second variable is in phase.

For the empirical analysis of time series correlations, the results are displayed as a map with several indicators or regions of investigation: brackets with eight possible directions expressed by the following arrows (←, →, ↑, ↓, ↘, ↙), black contours, two axes on the map, warm and cool colors. The →(←) symbols show a phase (out-of-phase) relationship or a positive (negative) correlation. In the case of the symbols ↑(↓) the leading effect of one or the other time series is shown. The symbol "↘" in wavelet analysis shows the phase relationship and positive joint movements of both time series. In this case, the first variable is lagged by π2. On the other hand, the "← " symbol confirms out-of-phase relationship and negative motion of both time series. Here in this case the second time series is lagged by π2.

The map shows a black curve that detects regions of coherence with 5 % significance. The next object of study is a white bell-shaped curve that detects a conical region of interest within the wavelet coherence.

The main motivating force for the use of wavelet analysis is then the following reasons. Firstly, this method can capture the time-variation trend in the commodity price, the interaction between lag levels, and the possible capture of time-series behavioral patterns. Furthermore, the non-stationary behaviour of signals, which is widespread in the case of commodities and agricultural commodities, plays a positive role for the use of the wavelet coherence method [88]. There is also a reason for using the wavelet coherence method to better capture structural changes in the time series. In contrast, methods that are based on autoregressive structure, such as Granger or dynamic causality, are not able to explain the simultaneous relationship between time and frequency in structural breaks in this case [76].

The main motivating force for the use of wavelet analysis is then the following reasons. Firstly, this method can capture the time-variation trend in the commodity price, the interaction between lag levels, and the possible capture of time-series behavioral patterns. Furthermore, the non-stationary behaviour of signals, which is widespread in the case of commodities and agricultural commodities, plays a positive role for the use of the wavelet coherence method [88]. There is also a reason for using the wavelet coherence method to better capture structural changes in the time series. In contrast, methods that are based on autoregressive structure, such as Granger or dynamic causality, are not able to explain the simultaneous relationship between time and frequency in structural breaks in this case [76].

## 3 Results

### 3.1 The statistical analysis of qualitative parameters of wood chip

The results of qualitative parameters of green wood chip samples in a major Polish region show the possibility of replacing fossil energy sources and at the same time how to reduce the carbon intensity in Poland. The analyses determine the quality parameters that are essential for direct energy recovery of wood chips in current combustion plants. This quality parameters depend mainly on the natural conditions in the given wood chip collection sites. One of these essential parameters is the calorific value of the wood chips.

**Fig. 2:**
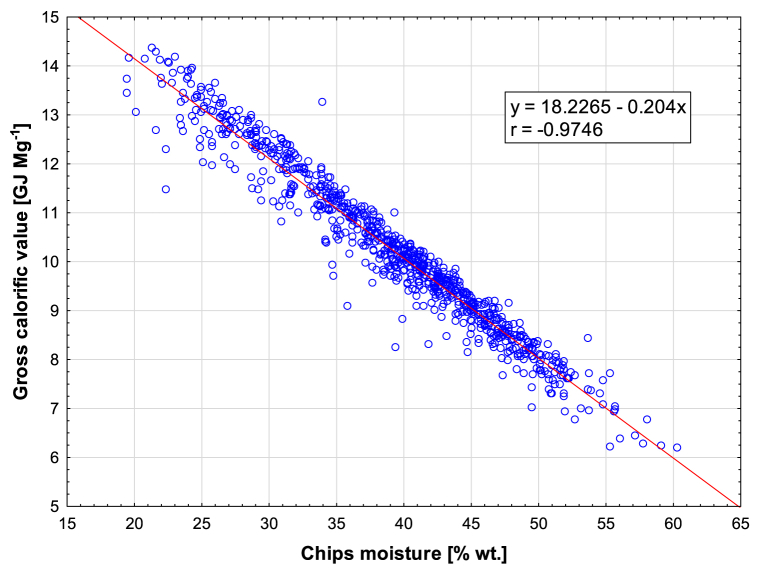
Dependence of calorific value on moisture content in green wood chip samples.

The resulting calorific values of the assessed green wood chips samples are shown in Fig. 2 as a function of the original moisture content. The results of the dependence show a linear trend with a high regression value. Over the whole measurement period, the average calorific value is 10.2 MJ kg^−1^, with the highest values of calorific value being reached in 2019, where the calorific value increased to 14.4 MJ kg^−1^. The lowest calorific value is reached in 2014 and 2017, where it is set at 6.2 MJ kg^−1^. The other calorific values are shown in Table 1 for each year of measurement. The greatest influence on the total calorific value in the samples is the moisture content of the green wood chips. The average moisture content was 39.24 % wt. over the whole period of interest, with the highest value of 60.3 % wt. being reached in 2017 and the lowest value was measured in 2019, when 19.5 % wt. was measured.

**Table 1:** Average values of calorific value (GJ Mg^−1^), moisture content (% wt.) and ash content (% wt.) for the study period in each year.

Year	*GCV* GJ Mg^−1^			*Ash* % wt.			*MC* % wt.		
	$\bar{X}$	SD	SE	$\bar{X}$	SD	SE	$\bar{X}$	SD	SE
2013	9.6	1.2	0.2	3.7	1.9	0.3	42.7	5.7	0.8
2014	10.5	1.7	0.1	3.4	1.9	0.2	38.5	7.7	0.7
2015	10.0	1.6	0.1	3.8	1.5	0.1	40.4	7.6	0.7
2016	10.0	0.9	0.1	4.0	1.7	0.2	40.2	4.2	0.4
2017	9.4	1.3	0.1	3.9	1.9	0.2	42.8	6.5	0.5
2018	10.2	1.8	0.2	3.7	2.0	0.2	39.0	8.5	0.8
2019	11.7	1.7	0.2	4.6	2.9	0.3	31.1	8.0	0.7

Note: $\bar{X}$ – mean; SD – Standard Deviation, SE – Standard Error, GCV – Gross calorific value; MC – Wood Chips Moisture Content.

**Fig. 3:**
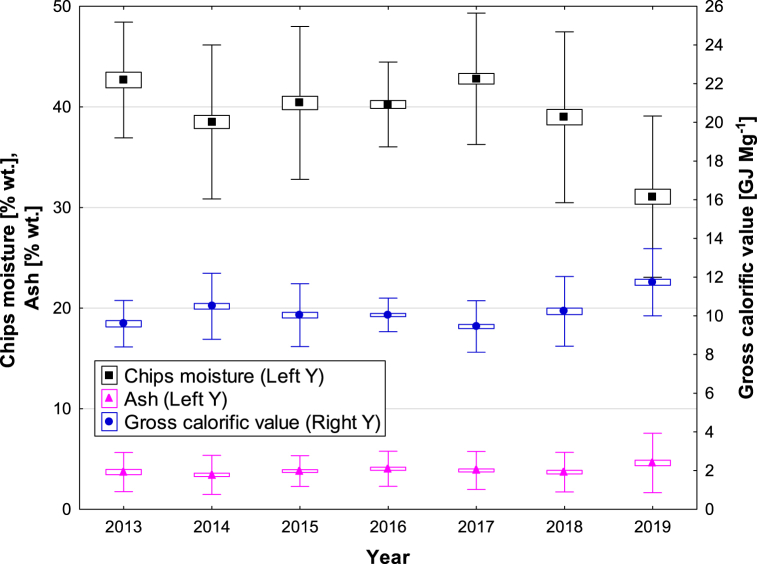
Moisture content, ash content and calorific value of green wood chip samples in the monitoring years 2013–2019.

Fig. 2 shows the dependence of calorific value on ash content. According to the regression analysis, the ash content has a very weak dependence on the calorific value of the samples. The moisture content values are shown in Fig. 3 and Table 1 for each year.

The next monitored non-combustible part of the fuel is the mass fraction of ash. The resulting values of ash content for each year are shown in Fig. 3 and Table 1 for each monitoring year. This is the measured data from the energy plant related to the analytical moisture content of the woodchips delivered from the forest to the plant's storage yard. Based on the measured moisture content and the calorific value, a settlement is made between the energy company and the supplier.

The amount of ash in the green wood samples averages 3.87 % wt. over the whole monitoring period. Maximum values exceeding up to 16 % wt. are reached in 2014 and 2019 and minimum values in 2017 and 2018 reached around 0.4 % wt. Such a high difference from the average values of ash is mainly due to the relative representation of moisture in the fuel, where in 2014 and especially in 2019, low moisture content in the samples is determined, therefore, there is a percentage increase in the other components in the fuel, and thus an increase in the average amount of ash in the samples in the years 2016 and 2019.

**Fig. 4:**
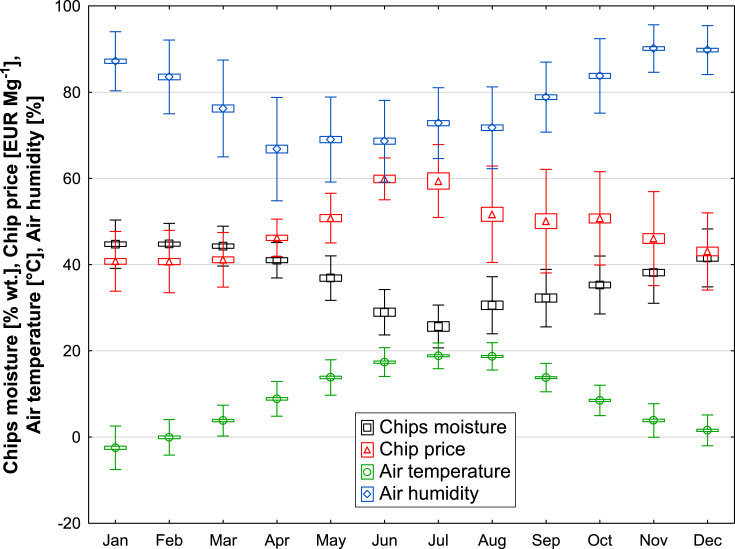
Moisture content, ash content and calorific value of green wood chip samples in individual months followed for the years 2013–2019.

The moisture contained in wood chips significantly influences the energy and economic parameters. In view of the year-round operation of wood chip management, it is necessary to assess these quality parameters also within the framework of the individual months of the year in relation to the atmospheric outdoor temperature and humidity conditions at the harvested sites. These conditions are monitored in Fig. 4 and Table 2 in the context of an important parameter such as the average monthly humidity in the sample and the price of wood chips for all the years monitored The minimum average moisture content is reached for the months of June and July, at an average of 27.3 % wt. moisture by weight in wood chips, but at the same time the price of wood chips increases (see Table 3). Conversely, in winter months such as December, January, and February, where the highest average monthly atmospheric moisture content of up to 89.8 % and the average monthly moisture content of wood chips of 43.7 % wt. is determined, the price of wood chips is higher than in the summer months.

**Table 2:** Average values of chip moisture (% wt.), air temperature (°C) and air humidity (%) for the monitored period in each month.

Month	*MC* % wt.			*AT* °C			*AH*%		
	$\bar{X}$	SD	SE	$\bar{X}$	SD	SE	$\bar{X}$	SD	SE
Jan	44.7	5.6	0.5	−2.5	5.1	0.4	87.2	6.9	0.5
Feb	44.8	4.8	0.5	0.0	4.1	0.3	83.5	8.5	0.7
Mar	44.3	4.6	0.5	3.8	3.6	0.3	76.2	11.2	0.8
Apr	41.0	4.1	0.6	8.9	4.0	0.3	66.8	12.0	0.9
May	36.9	5.1	0.7	13.8	4.1	0.3	69.0	9.8	0.7
Jun	28.9	5.3	0.9	17.4	3.3	0.2	68.6	9.5	0.7
Jul	25.7	5.0	1.1	18.8	3.0	0.2	72.8	8.2	0.6
Aug	30.6	6.6	1.0	18.7	3.2	0.2	71.7	9.5	0.6
Sep	32.2	6.7	1.0	13.8	3.3	0.2	78.9	8.1	0.6
Oct	35.3	6.7	0.7	8.5	3.5	0.2	83.8	8.6	0.6
Nov	38.2	7.1	0.7	3.8	3.9	0.3	90.1	5.5	0.4
Dec	41.6	6.7	0.7	1.5	3.6	0.3	89.8	5.7	0.4

Note: Descriptive statistics over month decomposition.

$\bar{X}$ – mean; SD – Standard Deviation, SE – Standard Error, MC – Wood Chips Moisture Content, AT – Air Temperature, AH – Air Humidity.

**Table 3:** Chip price in EUR GJ^−1^ and EUR Mg^−1^.

Month	Chips price EUR GJ^−1^			Chips price EUR Mg^−1^		
	$\bar{X}$	SD	SE	$\bar{X}$	SD	SE
Jan	4.46	0.57	0.05	40.77	6.93	0.66
Feb	4.47	0.58	0.06	40.71	7.25	0.72
Mar	4.51	0.53	0.06	41.12	6.33	0.66
Apr	4.73	0.48	0.07	46.20	4.36	0.61
May	4.74	0.39	0.06	50.79	5.77	0.83
Jun	4.90	0.20	0.04	59.89	4.87	0.87
Jul	4.64	0.43	0.10	59.39	8.44	1.89
Aug	4.33	0.66	0.10	51.67	11.17	1.63
Sep	4.25	0.60	0.09	50.08	12.02	1.72
Oct	4.54	0.69	0.07	50.73	10.83	1.05
Nov	4.37	0.65	0.07	46.03	10.90	1.13
Dec	4.38	0.59	0.07	43.05	8.96	1.00

Note: $\bar{X}$ – mean; SD – Standard Deviation, SE – Standard Error.

**Fig. 5:**
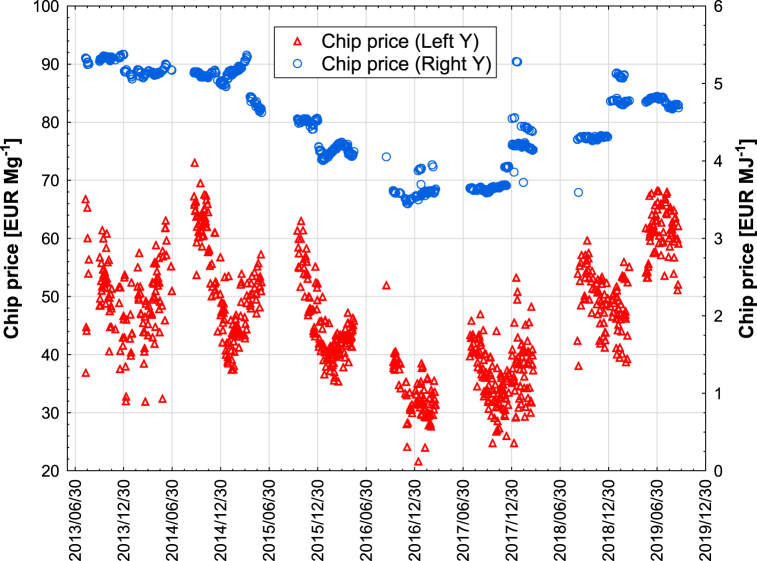
Change in wood chip price over time in Eur Mg^−1^ (left Y) and Eur GJ^−1^ (right Y) in following years (variance and pont chart).

Within the graphs, see Fig. 5 and Table 3 and it is possible to observe the relationship (monthly range) within the analyzed period between the price of wood chips in EUR and the calorific value in GJ Mg^−1^. Calculations can be performed to show that the price of woodchips in [EUR/GJ] has a lower coefficient of variation (CV = mean value/standard deviation) compared to the price of woodchips in [EUR/Mg]. The calorific values vary from 6 to 14 GJ Mg^−1^. The price ranges for these calorific values are defined by contract prices ranging from EUR 30 to EUR 80 for given months throughout the period. In the summer months, increasing calorific values result in an increasing contract price. In general, the correlation between calorific value and price follows approximately the same trend month by month over the observation period. For a more detailed price analysis of the spread across months, a graph is shown, see (Fig. 4, Table 3), where a strong effect of seasonality on the price of wood chips can be observed and the period when the price is identical. In 2016 and 2017, the lowest price was around 35 EUR Mg^−1^ (Fig. 4). The calorific value of wood chips is one of the quality parameters with a significant influence on the price.

The price analysis in terms of monthly distribution shows that the price fluctuates according to seasonal factors. In the spring months, there is an increasing trend in both the price of wood chips in GJ and Mg (Table 3). The highest prices were in June, July, and August. The price of wood chips shows a strong positive dependence on the calorific value.

### 3.2 Discrete wavelet transform (DWT)

**Fig. 6:**
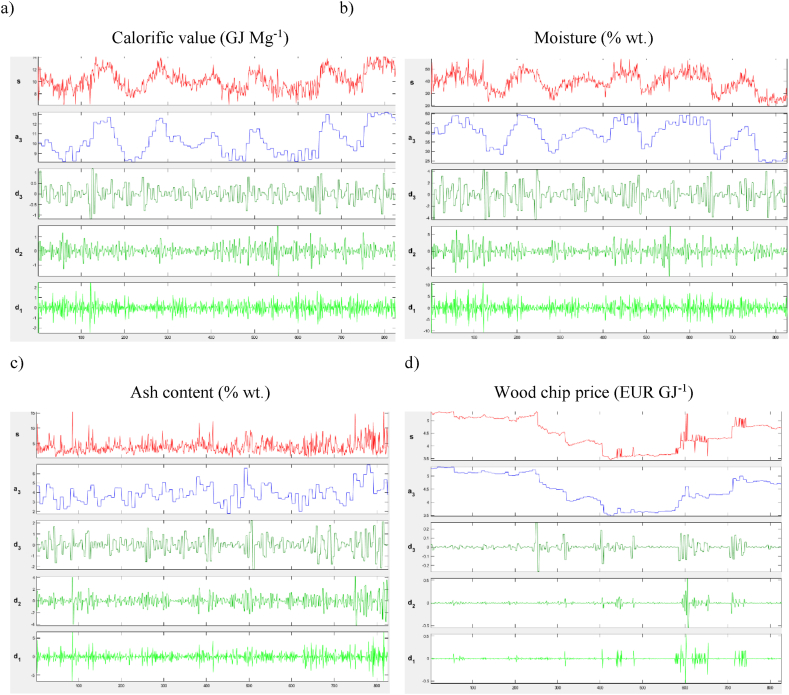
DWT: a – Calorific value (GJ Mg^−1^), b – Moisture (% wt.), c – Ash content (% wt.), d – Wood chip price (EUR GJ^−1^). Note: The x-axis shows the time series sequences corresponding to the period 2013 to 2019. The y-axis shows the individual decomposition components.

The investigated spectral transformations are based on wavelet analysis according to Daubechies, for more details see wavelet decomposition techniques. Thus, the wavelet decomposition is expressed by a third level decomposition: $s=a_{3}+d_{3}+d_{2}+d_{1}$, where s represents the time series, a_3_ the approximation of the given signal, and $d_{3},d_{2},d_{1}$ are then the individual components (coefficients) of the time scale. e results of discrete wavelet transform analysis of price DWT is a powerful tool for identifying scales, shifts and time variation in high frequency time series. This method is useful for decomposing the signal into greater detail and removing insignificant noise in pricing processes [89]. Closing prices of commodity futures are used to capture the strength of information in price movements, in contrast, for example, Yang et al. [90] use price returns for DWT analysis (Fig. 6). MATLAB software is used for the DWT analysis.

The DWT analysis decomposes the qualitative parameters signals to basic stones of lower levels. In Fig. 6 the signal decomposition of each parameter is shown.

The DWT analysis captures changes in the frequency of the parameters and their significant sources of fluctuation. For the qualitative parameters, i.e., Calorific value, Moisture content, Ash content, a stable trend of values can be observed within the period under study. Discrete transformations reveal the presence of seasonality depending on exogenous factors. Using the transformation, it is possible to detect the presence of dependence between the parameters Calorific value and Moisture content. The price of wood chips and its evolution over the period under consideration indicate unstable fluctuations that are influenced by exogenous factors. In terms of the short period of time within the decomposition parameters, significant fluctuations in calorific value and moisture content are evident. There is now direct research linked to decomposition using DWT among existing researches. The level of a_3_ levels in sub-figures (a), and (b) have a symmetric oscillation over the period. There is evidence of sensitivity to strong seasonal effects. Contrary, sub-figures (c), and (d) display in the case of a_3_ level lack of sensitivity on such effects. Among these parameters such ash price and ash content, see sub-figures (c) and (d) to the level of d_1_ the lower frequencies to 2–4 days in sense of stability there were rare fluctuations after 2017. Moreover, the implication of contractual wood chip price for predictability is limited due to the non-existence of autoregressive structure.

### 3.3 Wavelet coherence

**Fig. 7:**
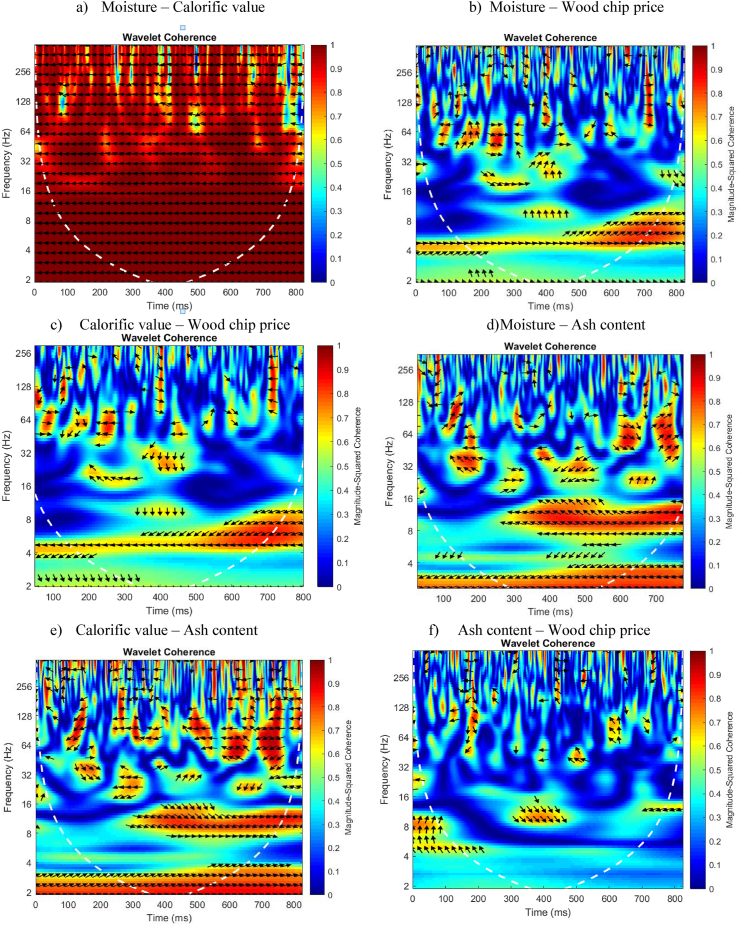
Wavelet coherence: a) Moisture – Calorific value, b) Moisture – Wood chip price, c) Calorific value – Wood chip price, d) Moisture – Ash content, e) Calorific value – Ash content, f) Ash content – Wood chip price. Note: The X-axis shows the time series data sequence of qualitative parameters corresponding to the period between 2013 and 2019. The Y-axis shows the frequency dependencies between.

The analysis of wavelet coherences within different frequencies (Hz) gives an overview of the possible correlations in the time-frequency domain between the different parameters (Fig. 7). In the first display of wavelet correlations, a very strong dependence between the qualitative parameters calorific value and moisture content can be observed. The red regions in sub-figure (a) have high level of correlation between moisture and calorific value with almost 99 %. The moisture in wood chips introduces high sensitivity on calorific character of wood chip. The cone of influence influenced by finite data set displays the strong correlation beyond it. The phase differences confirm the anticyclical phase sensitivity practically over the whole period of observation, respectively beyond seasonal effects. The signal of calorific value is lagged by π and vice versa. Areas mainly at higher frequencies, hence with short-term effects between parameters, indicate seasonal sources of qualitative fluctuations. These sources are independent of each other. It can also be inferred from the figure that at lower frequencies the correlation is even stronger, or the effect of a permanent strongly dependent relationship is confirmed. Other sub-figures (b), (c), (d), (e), and (f) display a weaker correlation over the period.

**Fig. 8:**
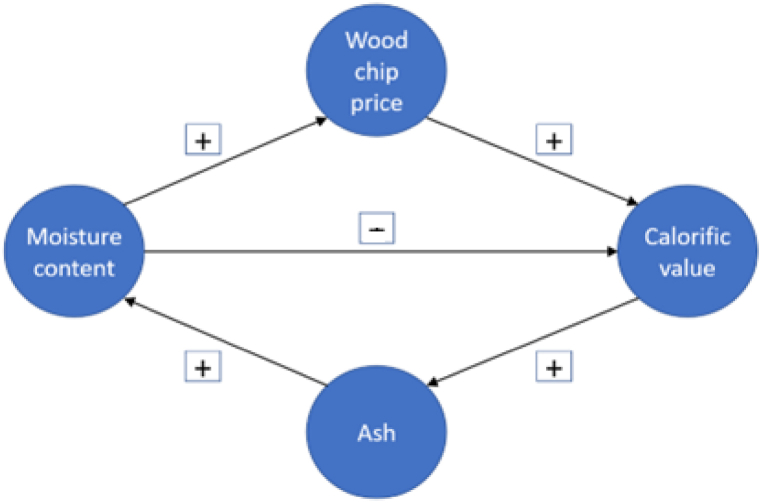
Cyclical and anticyclical patterns between calorific value, wood chip price and moisture and ash content.

The analysis of the correlation relationship at different frequencies within the moisture and contract price parameters provides a view of more separated areas of interdependence, see the following Fig. 8. The price nexus in relation to wood chip moisture is almost identical, but with an inverse correlation within the comparison with the calorific value parameter, see the following two figures. The phase differentials are purely inverse, the areas of correlation are identical, but not all the effects of the differentials are the same. In the second half of the observation period after 2015, at lower frequencies, there is a significant dependence between moisture and calorific value on the one hand and contract price on the other.

There is no clear correlation between moisture and ash parameters, especially in the short term. A negative (anti-cyclical) correlation can be observed at frequencies from 8 to 16 Hz. In the following display, the strong dependence between calorific value and moisture content of wood chips is confirmed.

The relationship between the parameter's ash content and wood chip price is the least demonstrated of all the quality parameters analyzed. The area where there is a relationship is around the middle of the period under study, i.e., 2016. There are effects within the conic influence but without interpretation. At the higher frequencies of 128–256 Hz in the short transient period, there is a more significant correlation between calorific value and ash content. Here, a significant seasonal effect between parameters is present. Based on the wavelet coherence, there are clear relationships between the quality parameters of wood chips. For better clarity, a plot of the phase differences detected based on the wavelet representation is shown, i.e., the direction of the interdependence of the quality factors. In the anticyclical sense there is linkage between moisture content and calorific value.

The analysis of in/out phase relationship shows that the moisture and ash content are out of phase in lower frequencies in the global area. There is an uncertainty with lead/lag relationship in the case of ash content and wood chip price. This linkage represents rare in/out of phase behaviour. Moreover, interesting findings support the evidence of anti-cyclically comovements of qualitative parameter values. Calorific value and moisture behave differently under conditions of high correlation. Both parameters depict high coherence at lower frequencies of 4–16 Hz. Furthermore, wood chip producers should consider other factors influencing the wood chip price rather ash content.

Based on the results, the research question is answered.

Answer to Q1: The inderdependency between qualitative parameters and price of wood chip exists on different levels of coherence and phase difference. The moisture and calorific value are based on the strong coherence with anti-phase nexus. The ash content and wood chip price represent a relationship with weaker coherence and unclear phase difference.

A cyclical and anticyclical (phase- and anti-phase following) relationship (Fig. 8) between calorific value, wood chip price and moisture content can be observed in the network graph of both positive and negative relationships, in-phase, or out-of-phase differences respectively. The calorific value is in anticyclical (out-of-phase) linkage with Moisture content. Other positive correlations are found between the parameters calorific value and ash content, and between ash content and moisture content. Moisture content also significantly influences the price of wood chips. The findings of this analysis also yielded a positive correlation between price and calorific value. When there is a relationship between an increase in calorific value and a higher price. The contractual price exhibits significant relationships among qualitative parameters due to strong dependence. Moreover, the linkage of ash content with wood chip price is not substantial. Producers of wood chips should consider other factors with more significant influence.

It is logical, that the moisture and calorific variables are strongly correlated because of the character of qualitative content. The results of strong linkage are in favour of research of Stolarski et al. [91]. According to the findings there is a significant role of transport and its type.

## 4 Discussion

The results of qualitative parameters of green wood chip samples in a major Polish region show the possibility of replacing fossil energy sources and at the same time how to reduce the carbon intensity in Poland. The analyses determine the quality parameters [42,92,93] that are essential for direct energy recovery of wood chips in current combustion plants, but also the economic parameters [54] sustainability of thermochemical technologies [[38], [39], [40]].

The moisture contained in wood chips significantly influences the energy and economic parameters. Content of moisture can be different more significantly according to type of fuel [44]. In view of the year-round operation of wood chip management, it is necessary to assess these quality parameters also within the framework of the individual months of the year in relation to the atmospheric outdoor temperature and humidity conditions at the harvested sites.

The magnitude of the set temperatures and the moisture content of the atmosphere affect the overall moisture content of the wood chips and thus affect the overall price of this commodity throughout the year, also in other areas [54]. Particularly during the summer months, the wood pulp dries out better.

The analyzed wood chips delivered to the power plant were characterized by significant compositional differences depending on where they were sourced, which, however, were averaged out by a significant amount of sampling and not distinguishing between individual deliveries at the power plant. It is natural to distinguish a large amount of needles and small (not yet woody) branches and bark in the logging residues that are chipped, in addition to wood. As reported by Nurek et al. [94], energy chips, in addition to pure wood, also contain various types of contaminants which is related to the technology of their extraction. During chipping, a certain amount of soil and sand is taken up along with the biomass, which directly affects the final share of ash and the energy value of the material a technical requirements for the combustion plant itself [45]. The wood chips delivered to the energy plant were characterized by an ash content of 3.4–4.6 % wt., which corresponds to the results reported by Gendek and Nurek [69], Gendek et al. [67] or Barontini et al. [95], but exceeds the values (0.4–2.9 % wt.) reported by Friedl et al. [96].

The seasonal change in wood chip moisture content per year has already been described by Gendek et al. [67]. Taking into account the change in moisture content of wood chips in the different months of the year, which directly affects their price (Fig. 4), it can be assumed that the best period for traders is the months from April to September. Unfortunately, in many cases, the summer season (June, July) is a period of maintenance work at many heating and power plants and the number of supplies is reduced. Entrepreneurs obtain wood chips during this time but deliver them to their own storage areas, where they are temporarily stored for about 2–3 months. Wood chips stored in piles tend to brew and moisten, so that in the month of August–September and their moisture content after delivery to the power plant increases. An additional factor in the increase in moisture content is atmospheric conditions associated with falling temperatures, rising humidity and precipitation.

In Poland, thermal power plants are the most important for energy production – both those that burn lignite and hard coal, but the pillar of the Polish energy sector is the latter [97]. Hydropower and alternative sources are clearly less important for electricity generation. Such a distribution of production results from the raw material and economic conditions. Poland, as a country rich in coal and lignite, naturally uses these raw materials to generate electricity. The production of electricity by coal mining also has a social and economic background, which hides the fear of miners' resistance to moving away from coal or the lack of financial resources to change the structure of energy production, e.g. nuclear. Economic changes and the need to change the approach to the use of raw materials (sustainable development) are causing a shift towards renewable energy sources, but power plants based on these sources are increasingly important [98].

Within the qualitative parameters analyzed with the detection of seasonal variations, a significant correlation between the observed parameters is found. The main challenge for the implementation of the European Commission's strategic plan under the Green Deal is to eliminate the weaknesses in the energy policy framework in Poland [99]. According to the results of the study, there is a need to significantly reduce the balance of coal materials for the energy sector. The adoption of the Polish energy policy until 2040 as a strategic plan addressing the dependence on fossil fuels in Poland in the long term is also key.

In their research, Hielscher et al. [100] demonstrated the potential for switching to non-fossil fuels in the UK, the Netherlands and Poland. The solution to the shift away from fossil fuels is only possible in breaking out of the policy interconnectedness between government and industry players. There are several areas where the interconnectedness of the fossil fuel demand relationship could be disrupted, firstly within an economic or resource approach where fuels that are more widely available are consumed, secondly to focus on the qualitative parameters of alternative energy commodities such as woodchips. By researching this aspect of the matter, it is possible in the long term to gradually move away from the significant need for fossil fuels and implement environmental policies within the European Union strategy.

According to Brauers and Oie [101], the biggest factors that hinder the growth of renewable energy in Poland are the share of workers in the coal industry and the economic dependence of regions. Furthermore, barriers to the development of alternative energy sources include the smaller scale of investment in this sector and the lack of government support for the competitiveness of the renewable energy market. In the political realm of determinants, the authors include the strong bargaining position of coal mine unions. According to the research results, the main obstacle to the development of renewable energy in Poland is the role of economic and political factors.

The influence of the European Union and its strategy of implementing the Green Deal could support and restructure the whole area of promotion and use of renewable energy sources, such as wood chips. Investigating the qualitative parameters of such sources could bring better practical applicability for easier implementation in the energy market.

The research question was confirmed according to time-frequency analysis. The connectedness between chip price and main qualitative parameters are significant. Wavelet analysis is very powerful tool to analyze price co-movements among different variables, for instance, the modification to Wavelet Local Multiple Correlation Method[102] or the crude oil sector with interconnectedness to energy and non-energy markets based on wavelets[103].

## 5 Policy implication

The empirical results have a significant impact on policy makers, such as local governments and producers.

The main issues regarding to findings.

- Quality monitoring must necessarily monitor the quality parameters of wood chips, especially moisture content and calorific value.
- Wood chip producers should focus on monitoring the relationship between price, calorific value, and moisture content rather than ash content.
- The calorific value of wood chips is lagged over moisture. This implies a time delay for producers to decide.
- The parameter of ash content and wood chip price show a more volatile seasonality effect than calorific value and moisture.

As the price of wood chips behave like a transmitter between calorific value and moisture there should be an establishment of policy for network and transportation among producers. The high volatility in seasonality is shown by wood chip price and ash content. Different frequencies show both long- and short-term cycles of qualitative parameter behaviour. The switching within different scales is important for production policy. The possible implications are considered for Polish region. Application overlap to other areas can be considered.

## 6 Conclusion

This paper focuses on the research area of alternative fuels, specifically wood chips in a major region in Poland that excels in this area of production within the country. Interrelationships in terms of permanent or transient relationships between different quality parameters such as calorific value, moisture content or ash content are considered. The contractual price of wood chips between processors is also considered separately. For the analysis of interconnectivity relationships, the method of wavelet coherences and discrete wavelet transforms is used to determine the individual components of the evolution of quality characteristics in the Polish region. The wood chip price and ash content show unstable seasonality effects. A strong positive cyclical relationship can be observed between moisture content, calorific value, and woodchip price. A high correlation is found between the parameters calorific value and moisture content, with almost 99 % interdependence. Based on the analysis, a region of mutual coherence is found at lower frequencies between the parameter's ash content, moisture content and calorific value. Here there is a long-term relationship, which is also influenced by seasonal effects, where there is no need for combustion during the summer months of the year. An unclear effect is shown for the parameters price and ash content. Here, a level of interdependence between values of 0.3–0.1 is found in most of the wavelet coherence map. In the short interdependence period, or at higher frequencies, significant relationships are found for the parameters calorific value and ash content, where seasonal ambiguous movements occur.

The research brings some interesting findings for producers of wood chips. For instance, a different behaviour of qualitative parameters. Firstly, the caloric value lags over moisture parameter. In other words, the relationship between these parameters is significant for considering of producers. The parameter of ash content depicts more less insignificant relationship between observed parameters. Moreover, wood chips producers should monitor wood chips price behaviour over other parameters. There is a limitation of this research regarding the local conditions of selected area in Poland. For future research, it is needed to consider other factors, such as price alternative energies, supply, and demand for energy. Furthermore, researchers should use the global or European dimension.

## CRediT authorship contribution statement

**Michal Čermák:** Methodology, Conceptualization. **Jitka Malaťáková:** Project administration, Formal analysis. **Jan Malaťák:** Supervision, Resources, Investigation. **Monika Aniszewska:** Visualization, Methodology, Data curation, Conceptualization. **Arkadiusz Gendek:** Methodology, Investigation, Data curation, Conceptualization.

## Declaration of competing interest

The authors declare the following financial interests/personal relationships which may be considered as potential competing interests:Michal Cermak reports financial support was provided by Czech University of Life Sciences Prague. Michal Cermak reports a relationship with Czech University of Life Sciences Prague that includes: employment. If there are other authors, they declare that they have no known competing financial interests or personal relationships that could have appeared to influence the work reported in this paper.
